# Prediction of uterine dehiscence using ultrasonographic parameters of cesarean section scar in the nonpregnant uterus: a prospective observational study

**DOI:** 10.1186/s12884-014-0365-3

**Published:** 2014-10-29

**Authors:** Michal Pomorski, Tomasz Fuchs, Mariusz Zimmer

**Affiliations:** Department of Gynecology and Obstetrics, Wroclaw Medical University, Borowska Street 213, 50-556 Wroclaw, Poland

**Keywords:** Cesarean section, Nonpregnant uterus, Uterine dehiscence, Transvaginal ultrasonography

## Abstract

**Background:**

Every year 1.5 million cesarean section procedures are performed worldwide. As many women decide to get pregnant again, the population of pregnant women with a history of cesarean section is growing rapidly. For these women prediction of cesarean section scar performance is still a serious clinical problem.

The purpose of the study was to assess whether the parameters of cesarean section scar in the nonpregnant uterus as determined using ultrasound can be used to predict uterine dehiscence in the next pregnancy.

**Methods:**

Starting in 2005, the study included 308 nonpregnant women with a history of low transverse cesarean section. The following ultrasonographic parameters of the cesarean section scar in the nonpregnant uterus were assessed: the residual myometrial thickness (RMT) and the width (W) and the depth (D) of the triangular hypoechoic scar niche. During 8 years of follow-up, 41 of these women were referred to our department for delivery. In all cases, a repeat cesarean section was performed and the lower uterine segment was assessed. Two independent statistical methods namely the logit model and Decision Tree analysis were used to determine the relation between the appearance of the cesarean section scar in the nonpregnat state and the performance of the scar in the next pregnancy.

**Results:**

The logit model revealed that the D/RMT ratio showed significant correlation with cesarean section scar dehiscence (*P*-value of 0.007). Specifically, a D/RMT ratio value greater than 1.3035 indicated that the likelihood of dehiscence was greater than 50%. The Decision Tree analysis revealed that a diagnosis of dehiscence versus non-dehiscence could be based solely on one criterion, a D/RMT ratio of at least 0.785. The sensitivity of this method was 71%, and the specificity was 94%.

**Conclusions:**

Assessment of the cesarean section scar in the nonpregant uterus can be used to predict the occurrence of scar dehiscence in the next pregnancy.

## Background

The number of women who have undergone cesarean sections (CS) increases by 1.5 million every year [1]. Thus, management of pregnant patients that have previously undergone CS has become routine in delivery rooms worldwide. However, we still don’t have sufficient predictive factors for individualized assessment of the risk of uterine rupture [2-4]. In addition, there are few tools for assessing the risk of uterine dehiscence, which itself is a strong risk factor for uterine rupture in vaginal birth after cesarean (VBAC) [5,6].

The method currently used to predict CS scar rupture is ultrasonographic measurement of the thickness of the lower uterine segment (LUS) in gestational week 36–38 as pioneered by Rozenberg et al. in 1996 [7]. The recent meta-analysis by Kok et al. supports the use of the LUS thickness for predicting uterine rupture during VBAC [8]. However, the heterogeneity of the methods used to measure LUS limit the clinical usefulness of this factor and does not allow the determination of universal cut-off values [8,9].

Recently, Naji et al. introduced a standardized approach for imaging and measuring CS scars during pregnancy and provided reference values for CS scar dimensions up to the 34^th^ week of gestation [10,11]. They also suggested that uterine scar rupture is associated with smaller residual myometrial thickness and with greater decrease in its thickness during the course of the pregnancy [12].

Another globally accepted option for assessing the CS scar is transvaginal ultrasonography of the nonpregnant uterus. When compared to the transabdominal approach, the proximity of the transvaginal probe to the pelvic organs enables obtaining high resolution images of the CS scar [13]. Several studies have assessed variations in the morphologic parameters of CS scars in the nonpregnant uterus in relation to the number of previously performed CS, clinical symptoms, flexion of the uterus, and maternal characteristics [14-17]. However, the principal question remained unanswered: Can the morphological parameters of CS scars in nonpregnant uterus be used to predict the integrity of the scar in the next pregnancy?

A search of Pubmed identified just one study by Olga Vikhareva Osser and Lil Valentin that compares the appearance of the scar in the nonpregnant uterus with the outcome of subsequent pregnancies [5].

The aim of the present study was to assess whether the ultrasound parameters of CS scars in the nonpregnant uterus can be used to predict uterine dehiscence in the next pregnancy.

## Methods

This long term, prospective study was performed in 2005–2013 in the Department of Gynecology and Obstetrics, Wroclaw Medical University, Poland. The study protocol was approved by the Ethics Committee of the Wroclaw Medical University.

Written informed consent was obtained from all patients. According to the written consent the patients were informed about the protocol of the study, about their participation in the study and that they are able to refuse to participate in the study at any time.

The protocol of the study was as follows:

1. Invitation to women with a history of low transverse CS to offer transvaginal ultrasound 6 weeks after CS.
2. Transvaginal ultrasound assessment of the CS scar in the nonpregnant uterus, including measurement of the following:
  - residual myometrial thickness (termed ‘RMT’).
  - width of the triangular hypoechoic scar niche (termed ‘W’).
  - depth of the triangular hypoechoic scar niche (termed ‘D’).
3. Assessment of the LUS (i.e. the CS scar) based on the operation protocol from the CS in the next pregnancy.

Ultrasound examinations were performed using a Medison SonoAce 8000SE or, beginning in 2007, using a Voluson 730 Pro (General Electric Medical Systems). Both ultrasound devices were equipped with a 4-9 MHz transvaginal probe that allowed proper visualization of the CS scar. All the ultrasonographic measurements were performed by single operator (M.P.).

To assess CS scars we used a procedure described in our previous publications [15,18,19]. For standardization purposes here we used the terms that were introduced by Naji et al. to describe the scar parameters [10].

The scar was identified in the sagittal transection of the uterus. The residual myometrial thickness (RMT) was defined as the distance between the tip of the hypoechoic triangle and the surface of the anterior uterine wall. Thus, RMT represents the thickness of the myometrial layer at the site of hysterotomy. In cases with completely healed CS scars, only this parameter was measured. In cases that had a hypoechoic triangular space in the lower part of the scar, the depth (D) and width (W) of the niche were also measured.

The depth of the hypoechoic triangle (D) was defined as the distance between the surface of the endometrial/endocervical layer of the posterior uterine wall to the tip of the hypoechoic triangle. The width (W) was defined as the distance between the proximal and distal parts of the myometrium of the anterior uterine wall measured at the surface of the endometrium/endocervix of the posterior uterine wall.

**Figure 1:**
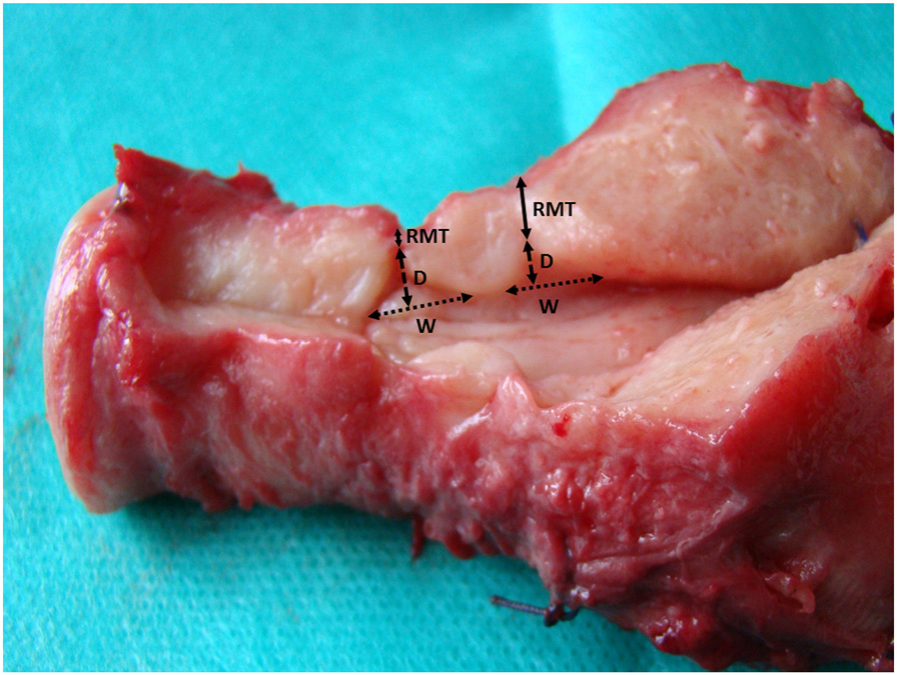
**The assessed cesarean section (CS) scar parameters on the uterus of a 50-year-old woman with a history of two lower transverse CSs.** RMT, residual myometrial thickness; W, width of the triangular hypoechoic scar niche; D, depth of the triangular hypoechoic scar niche.

**Figure 2:**
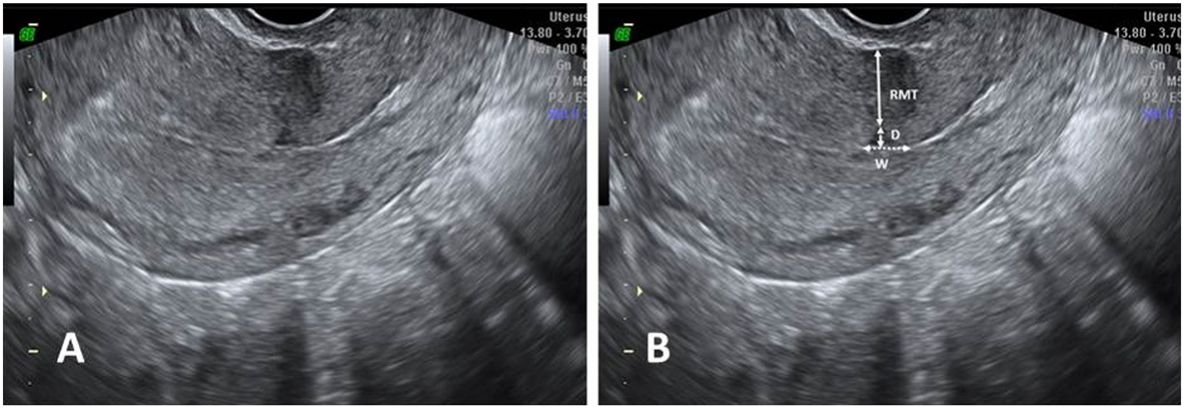
**The visualization of the cesarean section scar during transvaginal ultrasound.** **A** Presentation of the cesarean section scar **B** The assessed cesarean section scar parameters. RMT, residual myometrial thickness; W, width of the triangular hypoechoic scar niche; D, depth of the triangular hypoechoic scar niche.

Figure 1 shows the assessed CS scar parameters on the uterus of a 50-year-old woman with a history of two lower transverse CSs. The hysterectomy was performed due to endometrial hyperplasia. Figure 2A shows the visualization of the CS scar during transvaginal ultrasound. Figure 2B shows the assessed CS scar parameters.

After assessment of the CS scar in the non-pregnant uterus, the women were informed that according to the current knowledge, no clinical decisions could be based on the findings.

Women that became pregnant and were referred to our department for delivery were consulted to determine the best delivery method. VBAC was proposed to women that met certain criteria according to the recommendations of the Polish Gynecological Society [20].

Repeat CS was performed for women who did not accepted VBAC or who had medical indications for repeat CS. A standardized protocol that included assessment of the LUS was filled out by the obstetricians that performed the CS procedures. The three options for the assessment were:

1. Persistent LUS (persistent myometrium and perimetrium).
2. Uterine scar dehiscence (persistent perimetrium only).
3. Uterine scar rupture (no persistent tissue).

The obstetricians that assessed the scars were blinded to the results of the ultrasonographic assessment of the nonpregnant uterus.

Women with a history of ≥1 previous low transverse CS with single layer uterine closure were included in the study. Exclusion criteria were as follows: uterine malformations, CS performed before the 37^th^ week of gestation, a multiple pregnancy, incision other than a low transverse uterine incision, double-layer uterine closure, a history of puerperal infection, chronic corticosteroid administration.

### Statistical analysis

Statistical analysis was performed with R software, version 2.12.1 (2010-12-16).

Copyright (C) 2010 The R Foundation for Statistical Computing, ISBN 3-900051-07-0.

A *P* value less than 0.05 was considered statistically significant. Two independent statistical methods, namely the logit model and Decision Tree analysis were used to determine the relation between the appearance of the cesarean section scar in the nonpregnat state and the performance of the scar in the next pregnancy. The logit model is included in the library of basic procedures of the R software. It enables the estimation of the probability of CS scar dehiscence using explaining variables. The Decision Tree was constructed using the procedure ctree in the library party of the R software. This method provides a set of criteria (questions) that minimize the probability of a wrong decision based on patient’s characteristics.

## Results

Beginning in 2005, the study included 308 nonpregnant women in whom the ultrasonographic parameters of the CS scar were assessed 6 weeks after CS. Of this group, 43 women became pregnant and were referred to our department for delivery. Two women had twin pregnancies and were excluded from the study; thus, 41 women were included in the analysis. In all cases, a repeat CS was performed. There were no uterine scar ruptures in the studied group of women. The population was divided into two groups according to the intraoperative appearance of the LUS: persistent myometrium in the LUS (group 1) and uterine dehiscence (group 2).

In the group with persistent myometrium, 27 women (79.4%) had a history of 1 previous CS and 7 women (20.6%) had a history of 2 previous CSs. In the group with uterine dehiscence, 6 women (85.7%) had a history of 1 CS and one woman (14.3%) had a history of 2 previous CSs.

In this study, the term “first CS” refers to the CS that was performed 6 weeks before ultrasonographic assessment of the CS scar, and the term “second CS” refers to the CS in which there was intraoperative assessment of the scar. These terms are also used in the description of maternal characteristics.

Table 1 shows the characteristics of the studied groups.

**Table 1:** **Characteristics of the women in the studied groups**

	**Group 1**	**Group 2**	***P-value***
	**n = 34**	**n = 7**	
Age at first cesarean section (CS) (years ± SE)	30.1 ± 0.47	32.9 ± 1.34	0.09
Age at second CS (years ± SE)	33.1 ± 0.50	35.9 ± 1.18	0.06
Interval between the first and second CS (years ± SE)	2.9 ± 0.26	3.0 ± 0.44	0.91
Gestational age at first CS (weeks ± SE)	39.8 ± 0.23	39.9 ± 0.34	0.88
Gestational age at second CS (weeks ± SE)	38.9 ± 0.21	38.3 ± 0.29	0.09

Women in group 2 were older, but not significantly, than the women in group 1 at the time of first and second CS. There were no statistically significant differences between group 1 and 2 in terms of the interval between the CSs and gestational week at the time of the first and second CS.

Table 2 shows the indications for first and second cesarean section (CS) in groups 1 and 2.

**Table 2:** **Indications for first and second cesarean section (CS) in groups 1 and 2**

**Indications for CS**	**First CS group 1**		**First CS group 2**		**Second CS group 1**		**Second CS group 2**	
	**n = 34**	**%**	**n = 7**	**%**	**n = 34**	**%**	**n = 7**	**%**
Elective non-obstetrical indications	5	14.7	0		2	5.9	0	
Elective obstetrical indications	14	41.2	3	42.9	25	73.5	3	42.9
Emergency indications	15	44.1	4	57.1	7	20.6	4	57.1

In the nonpregnant uterus, transvaginal sonography allowed the CS scar to be visualized in all 41 women. A completely knit hysterotomy scar was identified in 11/41 cases (26.8%). In the remaining 30/41 patients (73.2%), a hypoechoic triangle, defined as the scar niche, was observed. In group 1, a completely healed CS scar was found in 10/34 cases (29.4%) and a scar with a hypoechoic triangle in 24/34 cases (70.6%). In group 2, the completely healed CS scar was visualized in 1/7 cases (14.3%) and a scar with a hypoechoic triangle in 6/7 cases (85.7%).

Table 3 shows the CS scar parameters and the W/RMT and D/RMT ratios for the entire study population and for groups 1 and 2. The D value and the D/RMT ratio value were significantly greater in group 2 than in group 1.

**Table 3:** **Mean values, standard errors, and** ***P*** **-values of cesarean section (CS) scar parameters and W/RMT and D/RMT ratios in the study population**

	**RMT ± SE**	**W ± SE**	**D ± SE**	**W/RMT ± SE**	**D/RMT ± SE**
Entire group	8.8 ± 0.6	5.2 ± 0.8	3.3 ± 0.4	0.83 ± 0.16	0.54 ± 0.10
Group 1	9.3 ± 0.7	4.7 ± 0.8	2.7 ± 0.4	0.68 ± 0.14	0.36 ± 0.07
Group 2	6.2 ± 1.3	6.9 ± 3.0	6.3 ± 1.2	1.58 ± 0.62	1.40 ± 0.39
*P* value	0.06	0.52	0.03	0.20	0.04

SE, standard error; W/RMT, the W/RMT ratio; D/RMT, the D/RMT ratio.

Table 4 shows the correlation between the risk of dehiscence and CS scar parameters and patient characteristics. Note the very low level of correlation between the incidence of CS scar dehiscence and the number of previous CSs, the interval between the first and second CS, and the gestational age at the time of first and second CS. There were higher correlation values between the RMT, W, and D values and the age of the women at the time of the CS. There was a significant correlation (0.60) between occurrence of CS scar dehiscence and the D/RMT ratio.

**Table 4:** **Correlation between the risk of dehiscence and the indicated variables**

	**deh**	**RMT**	**W**	**D**	**W/RMT**	**D/RMT**	**nCS**	**iCS**	**age1**	**GE1**	**GE2**
**deh**	**1.00** ^**a**^	**-0.29**	**0.16**	**0.48**	**0.33**	**0.60** ^**b**^	**0.04**	**0.02**	**0.35**	**0.02**	**-0.21**
**RMT**	**-0.29**	1.00^a^	-0.18	-0.27	-0.54^b^	-0.56^b^	-0.17	-0.23	-0.06	-0.23	-0.16
**W**	**0.16**	-0.18	1.00^a^	0.67^b^	0.84^a^	0.51^b^	0.12	-0.12	0.18	-0.06	-0.11
**D**	**0.48**	-0.27	0.67^b^	1.00^a^	0.60^b^	0.80^a^	0.18	-0.03	0.34	-0.03	-0.24
**W/RMT**	**0.33**	-0.54^b^	0.84^a^	0.60^b^	1.00^a^	0.75^a^	0.25	0.00	0.22	0.01	-0.07
**D/RMT**	**0.60** ^**b**^	-0.56^b^	0.51^b^	0.80	0.75^a^	1.00^a^	0.27	0.00	0.33	0.05	-0.12
**nCS**	**0.04**	-0.17	0.12	0.18	0.25	0.27	1.00^a^	0.08	0.05	-0.33	-0.17
**iCS**	**0.02**	-0.23	-0.12	-0.03	0.00	0.00	0.08	1.00^a^	-0.17	-0.10	-0.09
**age1**	**0.35**	-0.06	0.18	0.34	0.22	0.33	0.05	-0.17	1.00^a^	0.19	-0.06
**GE1**	**0.02**	-0.23	-0.06	-0.03	0.01	0.05	-0.33	-0.10	0.19	1.00^a^	0.44
**GE2**	**-0.21**	-0.16	-0.11	-0.24	-0.07	-0.12	-0.17	-0.09	-0.06	0.44	1.00^a^

^a^very high correlation; ^b^significant correlation. Deh: logic variable in which 1 = dehiscence, 0 = no dehiscence; RMT, residual myometrial thickness; W, scar width; D, scar depth; W/RMT, W/RMT ratio; D/RMT, D/RMT ratio; nCS, number of CS procedures; iCS, interval between first and second CS; age1, age of women at CS; GE1, gestational age at first CS; GE2, gestational age at second CS.

### Logit model analysis

**Figure 3:**
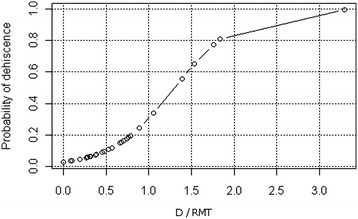
**The relationship between the probability of cesarean section scar dehiscence and the D/RMT ratio value.** D is the depth of the triangular hypoechoic scar niche, and RMT is the residual myometrial thickness.

The variables chosen for the logit model to determine the probability of CS scar dehiscence had correlation coefficients for which the variable deh was greater than 0.1 in terms of the absolute value (Table 5). All variables in the logit model for determining the probability of CS scar dehiscence had high *P*-values, making it necessary to perform a reduction of variables (Table 6). Stepwise backward selection of variables was used in the reduction. The reduced logit model for determining the probability of dehiscence revealed a very important variable: The D/RMT ratio showed a *P*-value of 0.007. Thus, the higher the D/RMT ratio, the greater the likelihood of CS scar dehiscence. An increase in the D/RMT ratio of 0.1 increases the chances of dehiscence by 30%. A D/RMT value greater than 1.3035 indicates that the likelihood of dehiscence is greater than 50%. Therefore D/RMT value greater than 1.3035 can be considered the first diagnostic criterion for the occurrence of CS scar dehiscence. The sensitivity of this method is 57%, and the specificity is 97%. Figure 3 shows the relationship between the probability of CS scar dehiscence and the D/RMT ratio value.

**Table 5:** **Coefficients, standard errors, and** ***P*** **-values of the logit model for probability of cesarean section scar dehiscence with all included variables**

**Variables**	**(Intercept)**	**RMT**	**W**	**D**	**W/RMT**	**D/RMT**	**age1**	**GE2**
Coefficient	23.29	0.22	0.92	-3.67	-1.34	5.62	0.28	-0.91
SE	29.86	2.55	4.38	6.72	2.73	4.80	0.23	0.82
*P*-value	0.44	0.93	0.83	0.59	0.62	0.24	0.22	0.26

SE, standard error; W/RMT, the W/RMT ratio; D/RMT, the D/RMT ratio; age1, the age of the women at the first CS; GE2, gestational age at the second CS.

**Table 6:** **Coefficients, standard errors, and** ***P*** **-values for the reduced logit model for probability of cesarean section scar dehiscence**

	**Coefficient**	**SE**	***P*** **-value**
(Intercept)	-3.55	0.96	0.0002
W/G	2.72	1.01	0.0070

SE, standard error; W/G, the W/G ratio.

### Decision tree analysis

**Figure 4:**
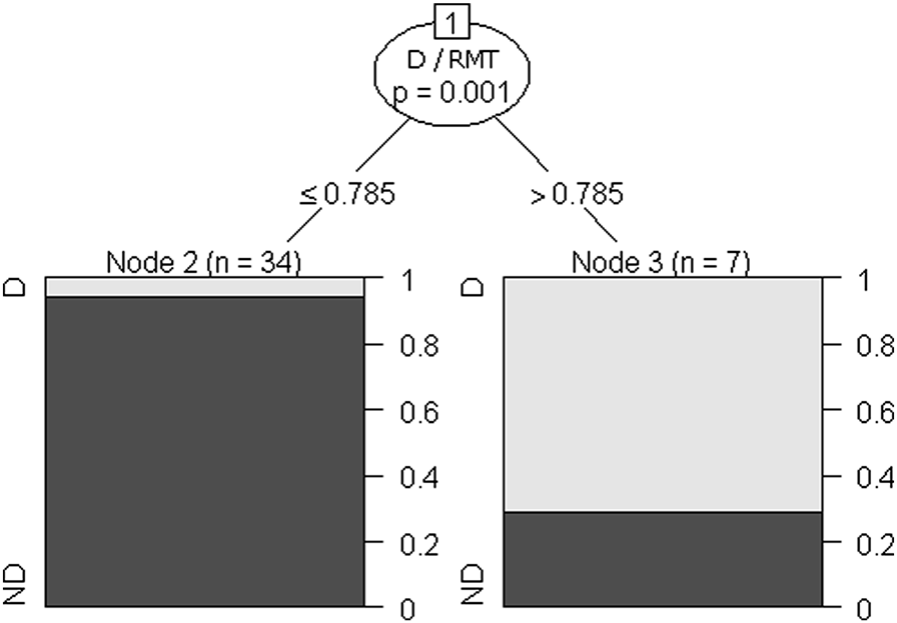
**Decision tree for diagnosis of cesarean section scar dehiscence using the D/RMT value, where D is the depth of the triangular hypoechoic scar niche, and RMT is the residual myometrial thickness.** D, dehiscence; ND, non-dehiscence.

The decision about whether a patient’s status indicates a high likelihood of CS scar dehiscence was based on a Decision Tree. The following variables were used: CS scar parameters; the W/RMT and D/RMT ratio values; the number of CSs; the interval between the first and second CS; the woman’s age at first CS; and gestational age at the time of first and second CS. This method revealed that the diagnosis should be based solely on one criterion - the D/RMT ratio value (Figure 4). The threshold value for the D/RMT ratio is 0.785. When the D/RMT ratio is greater than 0.785, CS scar dehiscence occurred; when the D/RMT ratio was lower than 0.785, there was no dehiscence. The sensitivity of this method is 71% and the specificity 94%. Compared with the method based on the logit model, this method has a higher sensitivity (+14%) and slightly weaker specificity (-3%).

It should be noted though that both methods show the usability of D/RMT ratio for assessment of the risk of CS scar dehiscence.

## Discussion

For the assessments of the CS scars in this study, we used a procedure that we described previously in 2007 [15]. As the number of patients included in the study group increased, we presented our results at the 18^th^ and 21^st^ World Congresses on Ultrasound in Obstetrics and Gynecology [18,19]. This method assess the same scar parameters as the standardized approach for measuring the CS scar in the pregnant uterus as described by Naji et al. in 2012 [10]. For this approach good interobserver agreement was proven [11]. The method used in this study has also similarities with the approach for measuring the CS scar in the nonpregnant uterus as described in the study by Osser and Valentin [5].

In this study the transvaginal approach was used to visualize the CS scar. The transabdominal approach was not used because in the nonpregnant uterus this procedure does not allow to obtain a good quality image of the CS scar region. The reasons for that are as follows: the long distance between the probe and the CS scar region, the localization of the CS scar region behind the pubic symphysis, interference by bowels and abdominal wall, and the need of full bladder [13].

In our study, the number of previous CSs, the interval between the primary and secondary CS, and gestational age at the time of the primary and secondary CS procedure showed no correlation with the incidence of CS scar dehiscence. Interestingly, there was a higher correlation for women that were older at the time of the CS. In the study by Naji et al. [12] the number of CS procedures had no impact on the CS scar dimensions over the course of pregnancy, but greater maternal age was associated with a greater increase in the width of the hypoechoic part of the scar.

Our results, based on the logit model and the Decision Tree, revealed that the only parameter that was useful for predicting CS scar dehiscence in the next pregnancy was the D/RMT ratio. In other words, the bigger the depth of the niche (D) and the smaller the thickness of the remaining myometrium (RMT), the greater the risk of CS scar dehiscence. Interestingly, none of the assessed CS scar parameters, when considered individually, was useful for predicting dehiscence. One possible explanation for the usefulness of the D/RMT ratio is that only the D and RMT values together represent the entire thickness of the uterine wall at the site of the scar.

In the study by Osser and Valentin [5] of women who delivered by CS, uterine dehiscence or rupture was found significantly less often in women with intact scars or with scars with a small defect (5.3%) compared to women with scars with a large defect (42.9%). There are several differences in the methodology used in our study versus the study by Osser and Valentin [5]. In their study, the defect was not measured but categorized as large or small based on the thickness of the RMT [5]. In addition, the time at which the CS scar was assessed was different: in the study by Osser and Valentin [5] the scar was assessed 6 to 9 months after CS, whereas in our study, it was assessed 6 weeks after CS. We chose to assess the scar 6 weeks after CS because at that time the scar is visible in all cases and, as it is the end of puerperium, the scar can be assessed easily in almost every patient during a routine visit.

The results of our study cannot be compared directly to the study by Osser and Valentin, however, it is notable that both studies found that the appearance of the CS scar in the nonpregnant uterus seemed relevant for predicting scar integrity in the next pregnancy.

The main limitation of our study is the relatively small group of patients who delivered after CS scar assessment. Of the 308 women, included in the study and followed up for 8 years, only 41 were referred to our department for delivery of a singleton pregnancy. The possible explanation for the reduction of the studied group is that many women had no future pregnancies or some of them decided to deliver in another hospital. Another limitation of our study is the lack of VBAC. The most common reason for repeat CS was refusal to sign the informed consent for VBAC. This is why we were not able to compare the parameters of the scar with VBAC outcome, but, on the other hand, next CS allowed direct assessment of the scar in every case.

There are several strengths of this study. First it is a long term, prospective study. Additionally, for the first time, CS scar parameters (including the dimensions of the scar niche) as measured in the nonpregnant uterus were used to assess the performance of the scar in the next pregnancy. Importantly, the myometrial part of the scar can give us information about scar integrity only when we also assess the dimensions of the hypoechoic part of the scar (which represents the not-knit myometrium). The proposed cut-off values indicating high risk of CS scar dehiscence are characterized by high specificity and sensitivity.

Our results provide support for the idea of including an assessment of the CS scar in the nonpregnant uterus together with currently used parameters (such as obstetric history, manual examination of the LUS, and ultrasound assessment of the CS scar in the pregnant uterus) in the process used to identify women who can safely have a VBAC versus patients that need to undergo elective CS procedures to avoid uterine rupture.

## Conclusions

Assessment of the CS scar in the nonpregant uterus can be used to predict the occurrence of cesarean section scar dehiscence in the next pregnancy. Additionally, worldwide there is a growing interest in surgical correction of the cesarean section scar defects [21,22]. As the assessment of the scar presented in this study is performed before the next pregnancy our results could be potentially useful for identification of women that will benefit from these procedures.

## Abbreviations

CS: Cesarean section
D: Depth of the triangular hypoechoic scar niche
LUS: Lower uterine segment
RMT: Residual myometrial thickness
VBAC: Vaginal birth after cesarean
W: Width of the triangular hypoechoic scar niche
